# Differential Regulation of *Syngap1* Translation by FMRP Modulates eEF2 Mediated Response on NMDAR Activity

**DOI:** 10.3389/fnmol.2019.00097

**Published:** 2019-05-09

**Authors:** Abhik Paul, Bharti Nawalpuri, Devanshi Shah, Shruthi Sateesh, Ravi S. Muddashetty, James P. Clement

**Affiliations:** ^1^Neuroscience Unit, Jawaharlal Nehru Centre for Advanced Scientific Research, Bengaluru, India; ^2^Institute for Stem Cell Science and Regenerative Medicine, Bengaluru, India; ^3^School of Chemical and Biotechnology, SASTRA Deemed University, Thanjavur, India

**Keywords:** FMRP, *Syngap1*, NMDA, eEF2, polysome, Autism Spectrum Disorder, Intellectual Disability, synaptoneurosome

## Abstract

SYNGAP1, a Synaptic Ras-GTPase activating protein, regulates synapse maturation during a critical developmental window. Heterozygous mutation in *SYNGAP1* (*SYNGAP1*^-/+^) has been shown to cause Intellectual Disability (ID) in children. Recent studies have provided evidence for altered neuronal protein synthesis in a mouse model of *Syngap1*^-/+^. However, the molecular mechanism behind the same is unclear. Here, we report the reduced expression of a known translation regulator, FMRP, during a specific developmental period in *Syngap1*^-/+^ mice. Our results demonstrate that FMRP interacts with and regulates the translation of *Syngap1* mRNA. We further show reduced *Fmr1* translation leads to decreased FMRP level during development in *Syngap1*^-/+^ which results in an increase in *Syngap1* translation. These developmental changes are reflected in the altered response of eEF2 phosphorylation downstream of NMDA Receptor (NMDAR)-mediated signaling. In this study, we propose a cross-talk between FMRP and SYNGAP1 mediated signaling which can also explain the compensatory effect of impaired signaling observed in *Syngap1*^-/+^ mice.

## Introduction

SYNGAP1 is a synaptic RAS-GTPase Activating Protein (SYNGAP1), which acts downstream of N-Methyl D-Aspartate Receptors (NMDAR), and negatively regulates RAS GTPase (Kim et al., 1998; Komiyama et al., 2002). Ca^2+^/Calmodulin-dependent Kinase II (CAMKII)-mediated phosphorylation of SYNGAP1 leads to rapid dispersion of SYNGAP1 from dendritic spine to the dendritic shaft leading to the activation of downstream signaling proteins in dendritic spines (Krapivinsky et al., 2004; Araki et al., 2015). Removal of SYNGAP1 from dendritic spines leads to increased activity of Extracellular Signal-Regulated Kinases (ERK) via RAS (Rumbaugh et al., 2006), which further allows insertion of α-amino-3-hydroxy-5-methyl-4-isoxazolepropionic acid Receptors (AMPAR) on the post-synaptic membrane (Zhu et al., 2002).

Studies using a mouse model have shown that *Syngap1*^-/+^ causes early maturation of dendritic spines in the hippocampus (Clement et al., 2012), and altered critical period of development in thalamocortical synapses (Clement et al., 2013). These studies have shown abnormal dendritic spine activity, and morphology coincided with an increased AMPAR/NMDAR-mediated currents during Post-Natal Day (PND)14–16 and 4–5 in the hippocampus, and thalamocortical neurons, respectively, that led to an altered critical period of plasticity in *Syngap1*^-/+^ mice. Consistent with its molecular function, studies from human patients have shown that loss-of-function mutations in *SYNGAP1* resulted in Intellectual Disability (ID), Autism Spectrum Disorder (ASD), and epilepsy (Hamdan et al., 2009, 2011; Rauch et al., 2012). All these studies suggest that SYNGAP1 is crucial for the development of neuronal connections during the critical period of development (Jeyabalan and Clement, 2016).

Recent studies using *Syngap1*^-/+^ mice and *Syngap1* knock-down in rat cultured cortical neurons demonstrated increased levels of basal protein synthesis in *Syngap1*^-/+^ as compared to WT (Wang et al., 2013; Barnes et al., 2015). The studies also suggested that SYNGAP1 modulates insertion of AMPARs at the post-synaptic membrane, thereby, regulating synaptic plasticity through protein synthesis (Rumbaugh et al., 2006; Wang et al., 2013). However, the molecular mechanisms for SYNGAP1-mediated regulation of protein synthesis, particularly during development, are unclear.

To regulate synaptic protein synthesis, SYNGAP1 may crosstalk with other translation regulators. One such potential candidate to consider is Fragile X Mental Retardation Protein (FMRP). Similar to *Syngap1*^-/+^ mice, *Fmr1* knock-out (KO) resulted in excessive levels of basal protein synthesis and altered dendritic spine structure and function (Huber et al., 2002). Additionally, a recent report showed exaggerated protein synthesis-independent mGluR-LTD (Metabotropic glutamate receptor-dependent long-term depression) in *Syngap1*^-/+^ (Barnes et al., 2015), which is another hallmark phenotype of FMRP associated synaptic deficits (Huber et al., 2002). Based on these findings, we hypothesized a possible cross-talk between SYNGAP1 and FMRP in regulating activity-mediated protein synthesis at the synapse. In this study, we have shown that FMRP level was altered during development, especially at PND21-23, in *Syngap1*^-/+^. Besides, FMRP interacts with and regulates the translation of *Syngap1* mRNA, and, thus, compensates for *Syngap1* translation in *Syngap1*^-/+^. These results may explain the impaired NMDAR-mediated signaling observed in *Syngap1*^-/+^.

## Materials and Methods

### Animals

C57/BL6 Wild-type (WT) and *Syngap1*^-/+^ mice were obtained from The Jacksons Laboratory^1^ (Kim et al., 2003) and bred and maintained in the Animal Facility, JNCASR, under 12-h dark and light cycle. This study was carried out in accordance with the principles of the Basel Declaration and recommendations of the Institutional Animal Ethics Committee (IAEC; Prof. Anuranjan Anand, Chairman). The protocol was approved by the Committee for Control and Supervision of Experiments on Animals (CPCSEA; Dr. K. T. Sampath, CPCSEA Nominee).

### Preparation of Hippocampal Slices

Acute brain slices were prepared from PND > 90 male and female WT and *Syngap1*^-/+^ mice. Mice were brought from the animal house and sacrificed by cervical dislocation, and the brain was dissected out. The brain was kept in ice-cold sucrose based artificial cerebrospinal fluid (aCSF; cutting solution) comprising of: 189 mM Sucrose (S9378, Sigma Aldrich), 10 mM D-Glucose (G8270, Sigma Aldrich), 26 mM NaHCO_3_ (5761, Sigma Aldrich), 3 mM KCl (P5405, Sigma Aldrich), 10 mM MgSO_4_.7H_2_O (M2773, Sigma Aldrich), 1.25 mM NaH_2_PO_4_ (8282, Sigma Aldrich) and 0.1 mM CaCl_2_ (21115, Sigma Aldrich). The brain was taken out of cutting solution and glued to the brain holder of the vibratome (Leica #VT1200), and 350 μm thick horizontal slices were prepared. Cortex and CA3 regions of the hippocampus were dissected out from each slice. All the slices were kept in slice chamber containing aCSF comprising: 124 mM NaCl (6191, Sigma Aldrich), 3 mM KCl (P5405, Sigma Aldrich), 1 mM MgSO_4_.7H_2_O (M2773, Sigma Aldrich), 1.25 mM NaH_2_PO_4_ (8282, Sigma Aldrich), 10 mM D-Glucose (G8270, Sigma Aldrich), 24 mM NaHCO_3_ (5761, Sigma Aldrich), and 2 mM CaCl_2_ (21115, Sigma Aldrich), in water bath (2842, Thermo Fisher Scientific) at 37°C for 45 min. Following recovery, slices were kept at room temperature (RT, 25°C) till the experiment completed. Post-dissection, every step was carried out in the presence of constant bubbling with carbogen (2–5% CO_2_ and 95% O_2_; Chemix, India). All measurements were performed by an experimenter blind to the experimental conditions.

### Extracellular Field Recordings

One slice at a time was placed on a bath chamber (Scientifica, United Kingdom) perfused with aCSF, and the temperature in the bath chamber was maintained at 33°C using in-line solution heaters (Warner Instruments, United States). Field excitatory post-synaptic potential (fEPSP) were elicited from pyramidal cells of CA1 area of stratum radiatum by placing concentric bipolar stimulating electrode (CBARC75, FHC, United States) connected to a constant current isolator stimulator unit (Digitimer, United Kingdom) at Schaffer-Collateral commissural pathway, and recorded from stratum radiatum of CA1 area of the hippocampus with 3–5 MΩ resistance glass pipette (ID: 0.69 mm, OD: 1.2 mm, Harvard Apparatus) filled with aCSF. Signals were amplified using Axon Multiclamp 700B amplifier (Molecular Devices), digitized using an Axon Digidata 1440A (Molecular Devices), and stored on a personal computer. Online recordings and analysis were performed using pClamp10.7 software (Molecular Devices). Stimulation frequency was set at 0.05 Hz. mGluR-LTD was induced by 5 min bath application of the Group I mGluR agonist (*S*)-3,5-dihydroxyphenylglycine (DHPG; Cat# 0805, Tocris, United Kingdom).

### Lysate Preparation

Brain lysates were prepared from Post-Natal Day (PND) 7–9, 14–16, 21–23, and adults (2–5 months). WT and *Syngap1*^-/+^ mice were sacrificed by cervical dislocation, brain was dissected out, and hippocampus was separated in cold Phosphate Buffered Saline (PBS) of pH 7.4 containing NaCl (137 mM, S6191, Sigma Aldrich), KCl (2.7 mM, P5405, Sigma Aldrich), Na_2_HPO_4_ (10 mM, 10028-24-7, Thermo Fisher Scientific), KH_2_PO_4_ (1.8 mM, GRM1188, HIMEDIA). The tissue was homogenized using RIPA buffer containing NaCl (150 mM, S6191, Sigma Aldrich,), Tris–Hcl (50 mM, Tris: 15965, Thermo Fisher Scientific; HCl: HC301585, Merck) pH 7.4, EDTA (5 mM, 6381-92-6, Thermo Fisher Scientific), Na-Deoxycholate (0.25%, RM-131, HIMEDIA), Triton X (1%, RM 845, HIMEDIA). Additionally, 1X Protease Inhibitor (P5726, Sigma Aldrich,), and 1X Phosphatase Inhibitor Cocktail 2 and 3 (P5726 and P0044, respectively, Sigma Aldrich) was added to the buffer to increase the stability of the lysate. Then, the homogenates were centrifuged at 16000 RCF for 30 min at 4°C. The samples were aliquoted and stored at -80°C. The supernatants were collected, and the protein was estimated using Bradford (5000006, Bio-Rad) or BCA (23225, Thermos Fisher Scientific) assay.

### SDS–PAGE and Western Blotting

The protein samples (50 μg in each lane) were electrophoresed on SDS (161-0302, Bio-Rad) Polyacrylamide (161-0156, Bio-Rad), 5% stacking gel for 30 min and 8% resolving gel (for FMRP, SYNGAP1, and PSD95) for ∼2 h or 10% resolving gel (for Phospho-eEF2, Total-eEF2, Phospho-ERK1/2, ERK1/2, and RPLP0) for ∼3 h. Overnight transfer at 20 V was done for the detection of Phospho-ERK1/2 (#9101, Cell Signaling Technology, 1:1000, raised in rabbit) and Total-ERK1/2 (#9102, Cell Signaling Technology, 1:750, raised in rabbit). Post-transfer, Ponceau staining was done, and Methanol was used as a fixative, and further washed with PBS. Blots were incubated with primary antibody (Phospho-ERK1/2, and Total-ERK1/2) for 4 h at room temperature (RT) in a shaker. For other proteins, transfer was done for 3 h at 80 V at 4°C onto Polyvinylidene Fluoride (PVDF) membrane (1620177, Bio-Rad) and blocked using 5% skimmed milk (GRM 1254, HIMEDIA) or 5% BSA (GRM105, HIMEDIA) in PBS for 1-h in Room Temperature (RT) at 25°C. BSA was used for blocking of all Phospho-Proteins. The blots were washed with 1% PBST (PBS+ Tween 20; GRM156 HIMEDIA) three times for 10 min each, and incubated with Primary Antibodies for FMRP (F4055, Sigma Aldrich, 1:1000 dilution, raised in rabbit), β-ACTIN (PA116889, Thermo Fisher Scientific, 1:15000 dilution, raised in rabbit), PSD95 (MA1-046, Thermo Fisher Scientific, 1:1000 dilution, raised in mouse), Phospho-eEF2 (Thr 56, 2331S, Cell Signaling Technology, 1:1000 dilution, raised in rabbit), Total-eEF2 (2332S, Cell Signaling Technology, 1:1000 dilution, raised in rabbit), and RPLP0 (ab101279, Abcam, 1:1000 dilution, raised in rabbit) overnight. After primary incubation, blots were washed with PBST thrice for 10 min each, then incubated with anti-Rabbit (1706515, Bio-Rad) or anti-Mouse (1706516, Bio-Rad) HRP conjugated Secondary antibody (1:10000 dilution). After subsequent washes with PBST, the blots were developed by a chemiluminescent method using ECL western clarity solution (1705060, Bio-Rad). Images were taken in Versa Doc (Bio-Rad), or ImageQuant (LAS 4000 from GE) or iBright FL1000 (Thermo Fisher Scientific) and merged using ImageLab version 5.2.1 and bands were quantified using ImageJ software.

### Immunoprecipitation

Hippocampus was dissected out from PND14-16 and 21-23 WT (littermates) and *Syngap1*^-/+^ (HET) as described earlier. Tissue was homogenized using Lysis buffer containing Tris–Hcl (50 mM, Tris: 15965, Thermo Fisher Scientific; HCl: HC301585, Merck), NaCl (150 mM, S6191, Sigma Aldrich), MgCl_2_ (5 mM, M8266, Sigma Aldrich), Dithiothreitol (DTT, 1 mM, 3483-12-3, Sigma Aldrich), NP40 (1%), RNase I (100 U/μl; Invitrogen, AM2294) and 1X Protease Inhibitor cocktail (P5726, Sigma Aldrich). All reagents were dissolved in Diethylpyrocarbonate (DEPC, D5758, Sigma) treated autoclaved water. Immunoprecipitation was done using anti-FMRP (F4055, Sigma Aldrich), Rabbit IgG (40159050MG, Millipore), and protein-G Dyna beads (10003D, Invitrogen). 30 μl of Dynabeads were equilibrated with lysis buffer, and further 200 μl of lysis buffer containing 5 μg of antibody was added to Dynabeads and incubated at RT for 1 h on a rotor at a slow speed. Afterward, the antibody solution was removed from the beads by placing the tube in the magnetic stand. Tissue lysate was added to the antibody bound beads and was incubated for 1-h at RT. The lysate was given five washes with lysis buffer. After the last wash, IP buffer was removed entirely, and the sample was eluted in either 1X Laemmli buffer (for protein detection) or Trizol (for RNA isolation). For the mRNA enrichment, mRNA copy number in the pellet was divided by mRNA copy number in the supernatant, unless otherwise mentioned.

### RNA Extraction and qPCR

Total RNA was extracted from the polysome fractions by Trizol (15596026, Thermo Fisher Scientific) method (For each sample three times the volume of Trizol was added) and the mRNAs were converted to cDNA using iScript cDNA synthesis kit (1708891, Bio-Rad). qPCR was performed for *Syngap1*, *Fmr1*, and *β-actin* using CFX384.

Real-Time System from Bio-Rad. Primers were designed and obtained from Sigma Aldrich, India. SYBR green was obtained from Bio-Rad (1725122). Ct values obtained from the reactions were converted to the copy number of the mRNA (Muddashetty et al., 2007, 2011), and the percentage of these copy numbers in each fraction was plotted for polysome experiments. mRNA copy number was derived using the Ct values from the standard curve. The equation for the standard curve was *y* = -1.44x+31.699; Here, y = average Ct value and EXP(x) was the copy number. List of primers used is mentioned below.

**Table:** 

Transcript	Forward sequence (5′3′)	Reverse Sequence (5′3′)
*Psd-95*	ATGGCAGGTT GCAGATTGGA	GGTTGTGA TGTCTGGGGGAG
*β-actin*	GGCTCCTAGC ACCATGAAGAT	AAACGCAGC TCAGTAACAGTC
*Syngap1*	CAACCGGAA GCTGGAAGAG	CATCAGCCT GCCAATGATGC
*Fmr1*	GCAGTTGGTG CCTTCTCTGT	GCTGCCTTGA ACTCTCCAGT


### Cell Culture and Transfection

HeLa cells were maintained in DMEM containing 10% FBS at 37°C in a 5% CO_2_ environment passaged using 0.05% trypsin-EDTA solution. Transfections were performed using lipofectamine 2000 (11668027, Thermo Fisher Scientific) as per the manufacturer’s protocol.

### Polyribosome Profiling Assay

Hippocampus was dissected out from PND21-23 and PND14-16 *Syngap1*^-/+^ (HET) and WT (littermates) as described earlier. Tissue was homogenized using Lysis buffer containing Tris–Hcl (200 mM, Tris: 15965, Thermo Fisher Scientific; HCl: HC301585, Merck), KCl (100 mM, P5405, Sigma Aldrich), MgCl_2_ (5 mM, M8266, Sigma Aldrich), Dithiothreitol (DTT, 1 mM, 3483-12-3, Sigma Aldrich), NP40 (1%), and 1X Protease Inhibitor cocktail (P5726, Sigma Aldrich). All reagents were dissolved in Diethylpyrocarbonate (DEPC, D5758, Sigma Aldrich) treated autoclaved water. Samples were aliquoted into two equal parts and treated with either of the protein synthesis inhibitors: Cycloheximide (CHX, 10 μg/ml, C7698, Sigma Aldrich) or Puromycin (1 mM, P9620, Sigma Aldrich). The lysates were kept at 37°C for 30 min and centrifuged at 4°C for 30 min at 18213 RCF. The supernatant was further loaded carefully on to the sucrose gradient prepared in polysome tubes. Sucrose (84097, Sigma Aldrich) gradient tubes were prepared 1-day before the day of the experiment. 15 to 45% gradients were made, and stored at -80°C. The supernatant was gently added to each polysome tubes (331372, BECKMAN COULTER), and ultra-centrifuged (Beckman, OptimaXL 100K) at 4°C at 39000 RPM for 1 h and 40 min. The tubes were then transferred to UV Visible spectrophotometer [Model: Type 11 Optical unit with reference Flowcell/No bracket, Serial No: 213K20162 at National Centre for Biological Sciences (NCBS)], and fractions were collected at A_254_ spectra using Fraction collector instrument (from TELEDYNE ISCO at NCBS). The bottom of the tube was pierced using a syringe attached to a pipe containing 60% sucrose, and the fractions were collected in 1.5 ml tubes. Total of 11 fractions was collected from each polysome tube, and these fractions were treated with SDS loading dye containing β-Mercaptoethanol (MB041, HIMEDIA) for immunoblotting or Trizol for RNA extraction and qPCR. SDS–PAGE was done for these fractions and immunoblotted for RPLP0 and FMRP.

### Synaptoneurosome Preparation and NMDA Stimulation

Hippocampus was dissected out from PND14-16, PND21-23, and PND30-35 mice as described earlier, and homogenized in 1000 μl of synaptoneurosome buffer containing NaCl (116.5 mM, S6191, Sigma Aldrich), KCl (5 mM, P5405, Sigma Aldrich), MgSO_4_ (1.2 mM, M7506, Sigma Aldrich), CaCl_2_ (2.5 mM, C5670, Sigma Aldrich), KH_2_PO_4_ (1.53 mM, GRM1188, HIMEDIA), Glucose (3.83%, G8270, Sigma Aldrich), 1X Protease Inhibitor Cocktail (P5726, Sigma Aldrich). Homogenate was filtered through 100 μm filter thrice (NY1H02500, Merck Millipore), and 11 μm filter once (NY1102500, Merck Millipore). The filtrate obtained was centrifuged at 1500 RCF for 15 min at 4°C. Pellet was resuspended in 1 ml synaptoneurosome buffer. NMDA receptor stimulation was done by applying NMDA (Final concentration 40 μM, M3262, Sigma Aldrich) for 1-, 2-, and 5-min, respectively, at 37°C in 350 RPM. 100 μM AP-5 was added to the sample preparation used for NMDAR-block experiments. The synaptoneurosomes prepared from the hippocampus of PND21-23 mice were aliquoted into three tubes. Two aliquots were treated with NMDA (40 μM), and NMDA+ AP-5 (100 μM, Cat#0105, TOCRIS) respectively. One tube was left untreated and considered as Basal level. Stimulation was done for 1 min at 37 °C in 350 RPM. After stimulation, the synaptoneurosomes were centrifuged at 11000 RPM for 21 s, and the pellet was resuspended in Lysis buffer followed by centrifugation at 18213 RCF at 4°C for 30 min. The supernatant was taken and denatured in loading dye containing SDS and β-Mercaptoethanol (MB041, HIMEDIA), and immunoblot assays were done.

### Statistics

**FIGURE 1:**
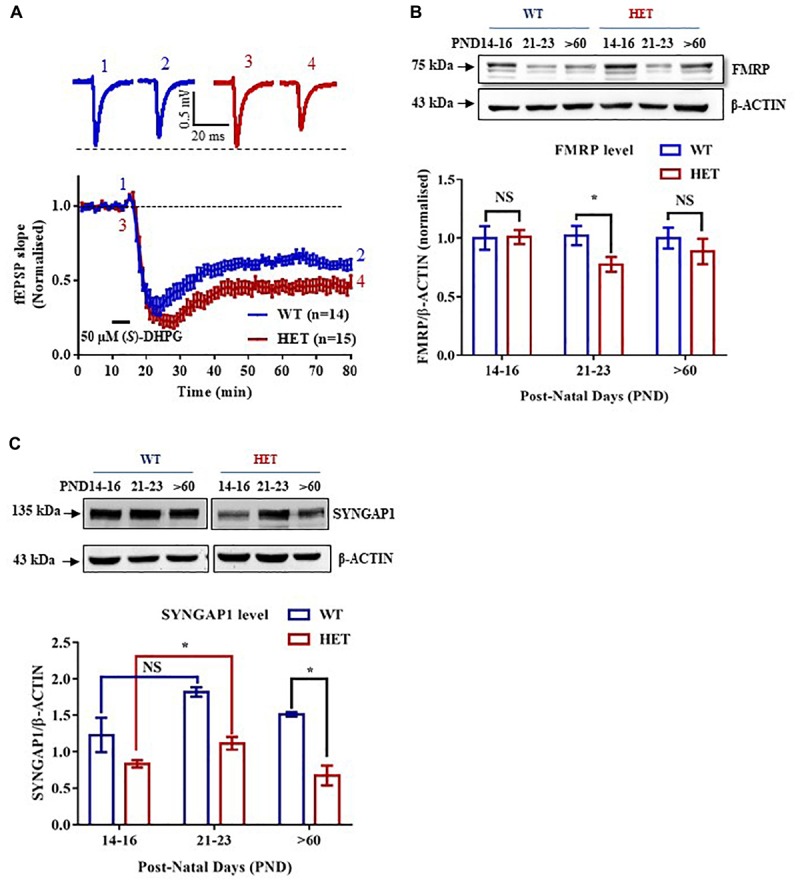
Altered expression of FMRP in the Hippocampus of *Syngap1*^-/+^ during development. **(A)** Application of 50 μm (*S*)-DHPG induced enhanced Group I mGluR mediated LTD in the Schaffer-Collateral pathway of adult (PND90) *Syngap1*^-/+^ (HET) compared to WT (WT) littermates. Sample traces obtained before and after the induction of LTD as indicated by time points (*top*). WT = 61 ± 3% LTD, *n* = 14; HET = 47 ± 4% LTD, *n* = 15; Unpaired Student’s *t*-test; ^∗^*p* < 0.05. **(B)** Representative immunoblot for FMRP level in the hippocampus during development (*top*). Pooled data of FMRP level normalized to β-ACTIN in the hippocampus during development, normalized to the level of WT (*below*). PND14-16 (WT: *N* = 10; HET: *N* = 8), PND21-23 (WT: *N* = 10; HET: *N* = 8), PND > 60 (WT: *N* = 8; HET: *N* = 10). ^∗^*p* < 0.05; Unpaired Student’s *t*-test. **(C)** Representative Immunoblots for SYNGAP1 during development (*top*). Histogram depicts SYNGAP1 level normalized to β-ACTIN in WT and HET at PND14-16, PND21-23, and PND > 60 (WT: *N* = 4; HET: *N* = 3). Bar graph shows increased SYNGAP1 level in HET during PND21-23 (WT: *N* = 4; HET: *N* = 5) when compared to PND14-16 (WT: *N* = 5; HET: *N* = 5) while no significant change was observed in WT. All WT and HET samples for individual age groups were run on the same gel. Two-way ANOVA; Unpaired Student’s *t*-test; ^∗^*p* < 0.05, NS, not significant.

All graphs were plotted using Graph Pad Prism 7 and Microsoft Excel 2016. Extracellular field recordings were performed and analyzed using Clampfit 10.7. Time course data shown in Figure 1A were plotted by averaging every 2 min. Example traces were those recorded for 1-2 min around the time point indicated. Error bars correspond to ± SEM (Standard Error of Mean). Unpaired Student’s *t-*test and 2-way ANOVA were performed to test for difference between groups and different age unless otherwise stated.

## Results

### Reduced FMRP Level During Development in *Syngap1*^-/+^

Studies have shown that Group I mGluR and NMDA receptors interact via Homer-Shank, thereby, regulating protein synthesis (Tu et al., 1999; Bertaso et al., 2010). To determine whether Group I mGluR activation in *Syngap1*^-/+^ resulted in altered protein synthesis and hippocampal pathophysiology similar to *Fmr1^-/y^*, Group I mGluR-mediated LTD (mGluR-LTD) was induced in the Schaffer-Collateral pathway in adult mice by bath applying 50 μm (*S*)-DHPG, Group I mGluR agonist, for 5 min. We observed significantly increased mGluR-LTD in *Syngap1*^-/+^ mice (*Syngap1*^-/+^ referred as HET in Figures; 47 ± 4% LTD) as compared to their WT littermate controls (61 ± 3% LTD; *p* = 0.012; Figure 1A). This result suggests that mGluR-LTD in *Syngap1*^-/+^ is similar to *Fmr1^-/y^* at PND25-32 as shown earlier by Barnes et al. (2015). Our data further showed that abnormal signaling during early stages of development, in fact, continues throughout adulthood (PND90) that may explain the impaired cognitive and social behavior observed in adults. Therefore, we hypothesized that expression of FMRP might be altered during different neurodevelopment stages, including adulthood.

We studied the expression of FMRP in the hippocampus of WT and *Syngap1*^-/+^ mice during different stages of development, starting from PND7-9 to 2-5 months of age. Using quantitative immunoblotting, we observed that FMRP level (normalized to β-ACTIN) was reduced in *Syngap1*^-/+^ mice (0.775 ± 0.06) as compared to WT in PND21-23 (1.00 ± 0.07; *p* = 0.033; Figure 1B) but not in other age groups. FMRP expression profile in WT shows that FMRP level decreases as age increases (Supplementary Figure S1A). Previous studies have shown that reduced SYNGAP1 expression during development led to altered synaptic transmission in *Syngap1*^-/+^ mice (Vazquez et al., 2004; Clement et al., 2012). To study whether reduced level of FMRP is compensating for the altered SYNGAP1 level in *Syngap1*^-/+^ mice, expression of SYNGAP1 in WT and *Syngap1*^-/+^ mice was quantified as shown in Figure 1C and Supplementary Figure S1B (Genotype: *p* < 0.0001). Upon further analysis, we found that the SYNGAP1 level was increased during PND21-23 (1.12 ± 0.09) compared to PND14-16 in *Syngap1*^-/+^ (0.83 ± 0.05; *p* = 0.0236; Figure 1C), and no statistical difference was observed in adults (>PND60). In contrast, the level of SYNGAP1 was not altered significantly between PND21-23 (1.82 ± 0.06) and PND14-16 (1.33 ± 0.08) in WT mice (*p* = 0.0863; Figure 1C). In our study, we considered β-ACTIN as an internal control for normalization. However, β-ACTIN polymerisation-depolymerisation could be modulated by FMRP. Thus, we validated our results using GAPDH as a loading control that showed an expression profile for FMRP, and SYNGAP1 in WT similar to quantification performed with β-ACTIN (Supplementary Figures S1A,B).

### FMRP Interacts With *Syngap1* mRNA and Regulates Its Translation

**FIGURE 2:**
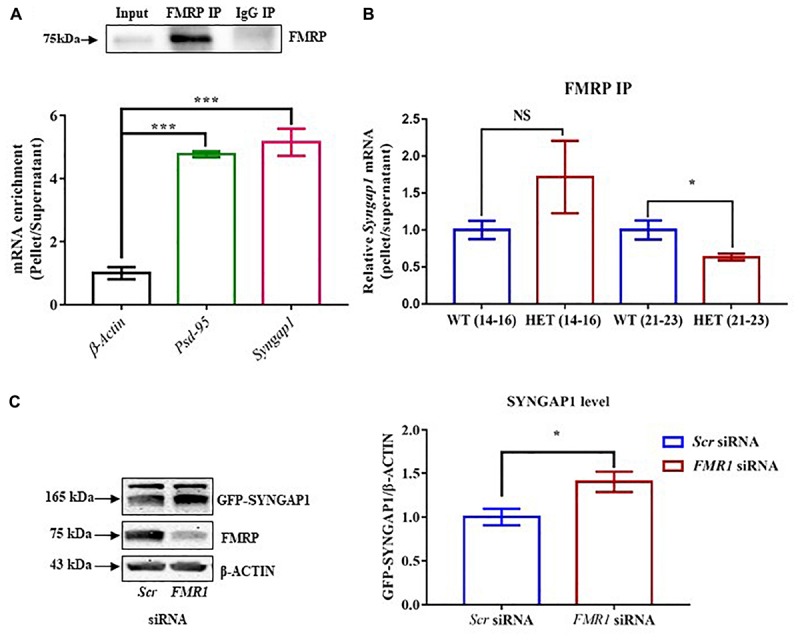
FMRP regulates *Syngap1* mRNA translation. **(A)** Immunoblot for FMRP following FMRP-IP and IgG-IP (*top)*. Bar graph showing relative *Syngap1*, *Psd95* mRNA enrichment in FMRP IP pellet compared to Supernatant after normalizing to *β-Actin* (WT: *N* = 3; Below). Enrichment was calculated by the given formula: 2^-(dCtFMRPIP)^; dCt = Ct (pellet) – Ct (Supernatant); One-way ANOVA followed by Dunnett’s multiple comparisons test. ^∗∗∗^*p* < 0.0001. **(B)** The bar graph shows relative *Syngap1* mRNA enrichment in FMRP IP pellet compared to supernatant from hippocampus at PND21-23 (WT: *N* = 5; HET: *N* = 4), and PND14-16 (WT: *N* = 4; HET: *N* = 3) normalized to WT. Unpaired Student’s *t*-test; ^∗^*p* < 0.05; NS, not significant. **(C)** Representative immunoblot for SYNGAP1, FMRP, and β-ACTIN showing knock-down of FMRP leads to increase SYNGAP1 expression in Hela (*left*). The quantified bar graph shows an increase in the level of GFP-SYNGAP1 expression in the cells treated with *FMR1* siRNA compared to *Scr* siRNA treatment (*right*). Unpaired Student’s *t*-test; ^∗^*p* < 0.05.

FMRP is a known regulator of synaptic translation (Osterweil et al., 2010). A previous study using HITS-CLIP has reported *Syngap1* as one of the mRNAs regulated by FMRP (Darnell et al., 2011; Darnell and Klann, 2013). G-quadruplexes are one of the structures present in RNA which could be recognized by FMRP (Darnell et al., 2001). Bioinformatics analysis using Quadruplex forming G-Rich Sequences (QGRS) Mapper predicted the presence of multiple G-quadruplexes structures with high G-Score in *Syngap1* mRNA (Supplementary Figure S1C). Besides, G-quadruplex forming residues were found to be conserved among mice, rat, and human *Syngap1* mRNA (Supplementary Figure S1C). To further confirm the interaction of FMRP with *Syngap1* mRNA, we performed FMRP immunoprecipitation from mouse hippocampal lysates to investigate the enrichment of *Syngap1* mRNA by qPCR. We observed a ∼5-fold enrichment of *Syngap1* mRNA relative to *β-actin* mRNA in FMRP-IP pellet over supernatant (5.15 ± 0.43, *p* = 0.0009; Figure 2A, Supplementary Figure S2A). *Psd-95* mRNA, a known FMRP target mRNA (Muddashetty et al., 2011) showed a significant 4.5-fold enrichment compared to *β-actin* mRNA (4.77 ± 0.09; *p* = 0.0001; Figure 2A and Supplementary Figure S2A), which we used as a positive control. These results demonstrated that FMRP interacts with *Syngap1* mRNA.

We further asked whether the interaction between FMRP and *Syngap1* mRNA is altered during development, especially in PND14-16 and 21-23. We did not find a statistical significance in PND14-16 (*p* = 0.28; *Syngap1*^-/+^ = 2.3 ± 0.66; WT = 1.0 ± 0.1; Figure 2B). Whereas, the interaction between FMRP and *Syngap1* mRNA was significantly decreased in *Syngap1*^-/+^ at PND21-23 (*p* = 0.045; 0.63 ± 0.04; Figure 2B) compared to WT (1.0 ± 0.13). We did not observe any change in the interaction of *Psd-95* mRNA with FMRP at any of these age groups (PND14-16: *p* = 0.44; *Syngap1*^-/+^ = 1.297 ± 0.34; WT = 1.0 ± 0.2; PND21-23: *p* = 0.24; *Syngap1*^-/+^ = 0.8347 ± 0.12; WT = 1.0 ± 0.07; Supplementary Figure S2B) To validate these findings further, we overexpressed *GFP*-*Syngap1* in Hela cells followed by knock-down of *Fmr1* (decreased expression of FMRP; Supplementary Figures S2C–E). We have shown that a reduction in FMRP led to an increase in GFP-SYNGAP1 (*p* = 0.01; *Scr* siRNA 0.58 ± 0.05; *FMR1* SiRNA 0.82 ± 0.067; Figure 2C). These results demonstrated that FMRP not only interacts with *Syngap1* mRNA but also regulates its translation. On the basis of our data, we speculate that reduced interaction between FMRP and *Syngap1* mRNA in *Syngap1*^-/+^ at PND21-23 might lead to increased SYNGAP1 level as observed earlier.

### *Syngap1* mRNA Translation Differentially Regulated in *Syngap1*^-/+^

**FIGURE 3:**
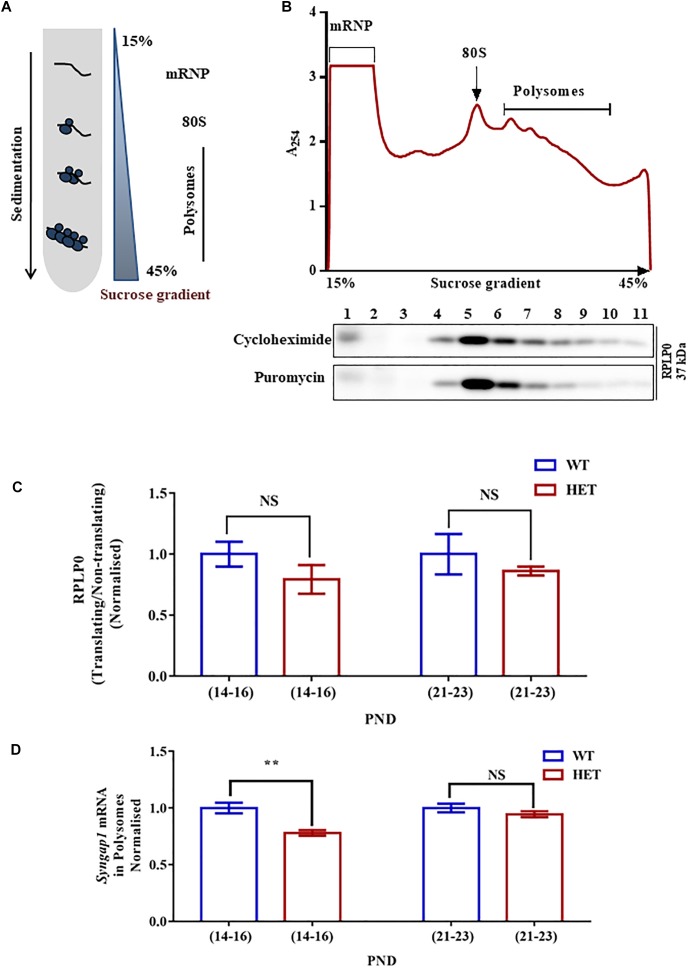
Altered *Syngap1* mRNA distribution in translating polysomal fractions of HET. **(A)** Schematic diagram depicting the sucrose gradient method used for polyribosome profiling (translation assay). **(B)** Polyribosome profile obtained from Cycloheximide treated hippocampal lysate during PND14-16 in HET (*top*). Representative immunoblots for RPLP0 distribution in Cycloheximide and Puromycin treated polysome during PND14-16 (*below*). **(C)** Bar graph shows RPLP0 distribution in Translating/Non-translating fractions during PND14-16 (WT: *N* = 5; HET: *N* = 3; *p* = 0.22) and PND21-23 (WT: *N* = 4; HET: *N* = 4; *p* = 0.14). Unpaired Student’s *t*-test was done for both age groups. NS, not significant. **(D)** *Syngap1* mRNA distribution in polysome in HET normalized to WT during PND14-16 (WT: *N* = 4; HET: *N* = 6; *p* = 0.0018) and PND21-23 (WT: *N* = 3; HET: *N* = 3; *p* = 0.3033); ^∗∗^*p* < 0.01; Unpaired Student’s *t*-test.

To further understand the mechanistic details of the compensatory increase in SYNGAP1 levels during PND21-23 in *Syngap1*^-/+^, we analyzed *Syngap1* mRNA translation status at PND14-16 and PND21-23. We studied translation by Polysome profile (Figure 3A) from hippocampal lysates of WT and *Syngap1*^-/+^ mice at PND14-16 and PND21-23 (Muddashetty et al., 2007). Based on the A_254_ traces from cycloheximide-treated samples, Figure 3B showed the distinct peaks corresponding to mRNP, monosome, and polysomes, respectively. A_254_ traces between WT and *Syngap1*^-/+^ mice did not show any significant difference, suggesting that the global translation in hippocampus might be unaffected in *Syngap1*^-/+^ mice at PND14-16 and PND21-23. Further, immunoblots for Ribosomal large subunit protein, RPLP0, has shown a shift in puromycin treated samples as puromycin disassemble the ribosome from translating mRNA (Figure 3B), along with a shift in *β-actin* mRNA (Supplementary Figure S3A). Additionally, as a quality check for RNA integrity, we checked for 18S rRNA distribution in the polysomal fractions of cycloheximide and puromycin treated samples. Our results showed a shift of rRNA toward non-polysomal fractions upon puromycin-treatment as compared to cycloheximide-treatment (Supplementary Figures S3B,C). In our experiments, fraction numbers 1 to 6 and 7 to 11 were considered as non-translating fractions and translating fractions or polysome (puromycin-sensitive) respectively, on the basis of RPLP0, 18S rRNA, and *β-actin* mRNA distribution (Supplementary Figures S3A–C). As a control, distribution of *β-actin* mRNA in translating pool was quantified, and no significant difference was observed between WT and *Syngap1*^-/+^ at PND14-16 (WT = 89.9 ± 3%; *Syngap1*^-/+^ = 83.6 ± 2.9%; *p* = 0.2039) and PND21-23 (WT = 97.9 ± 0.6%; *Syngap1*^-/+^ = 89.8 ± 3.8%; *p* = 0.0697; Supplementary Figure S3D). Next, we estimated the RPLP0 distribution in translating/non-translating fractions of WT and *Syngap1*^-/+^ during PND14-16 (WT = 1.06 ± 0.18, *Syngap1*^-/+^ = 0.71 ± 0.15, *p* = 0.22) and PND21-23 (WT = 1.27 ± 0.21, *Syngap1*^-/+^ = 0.83 ± 0.17, *p* = 0.14), suggesting no significant change in the distribution of RPLP0 (Figure 3C and Supplementary Figure S3E). To understand the translation status of *Syngap1* mRNA during PND14-16 and PND21-23, we quantified *Syngap1* mRNA present in translating fraction by performing quantitative PCR from RNA isolated from both non-translating (Fractions 1-6) and translating fractions (Fractions 7-11). However, a significant reduction in translating *Syngap1* mRNA in *Syngap1*^-/+^ mice during PND14-16 compared to WT was observed (WT = 84.32 ± 4%; *Syngap1*^-/+^ = 65.77 ± 2%; *p* = 0.0018; Figure 3D). On the contrary, this difference was absent during PND21-23 (WT = 92.9 ± 3.5%; *Syngap1*^-/+^ = 87.9 ± 2.5%; *p* = 0.3033). Our data demonstrate that an increase in *Syngap1* mRNA translation leads to the corresponding increase in SYNGAP1 level in PND21-23 when compared to PND14-16 in *Syngap1*^-/+^. As a control, we treated the samples with Puromycin, and it did not show distinct polysome peaks indicating disassembly of ribosomes from the mRNA (Supplementary Figure S3F).

### Reduced FMRP in Polysome at PND21-23 in *Syngap1*^-/+^

**FIGURE 4:**
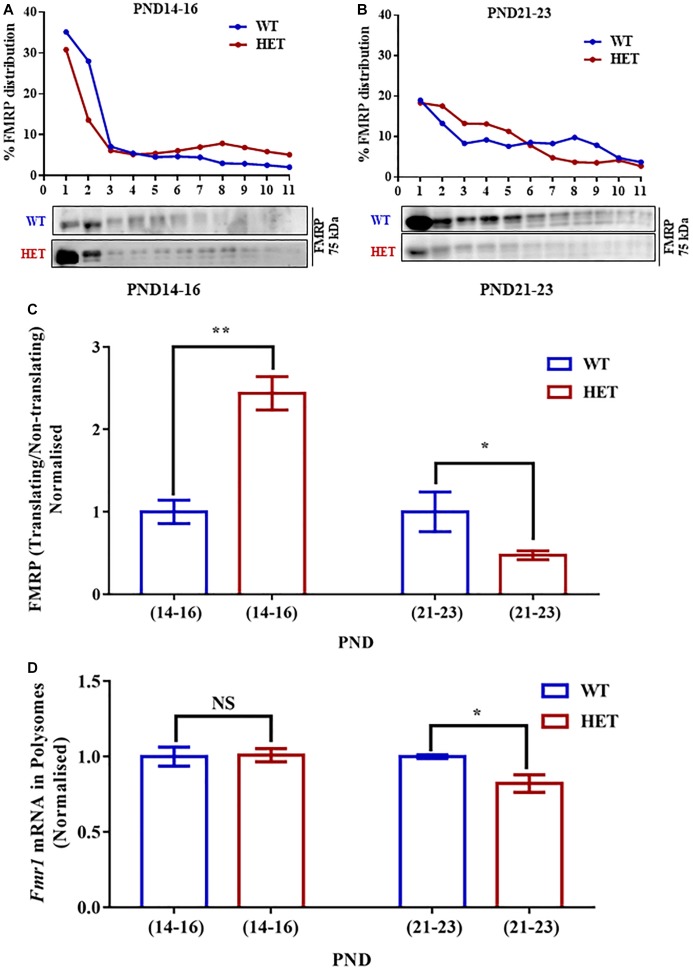
Decreased *Fmr1* mRNA and FMRP distribution in translating fractions of polysomes in HET during PND21-23. **(A)** Representative line graphs showing percentage FMRP distribution in polysomes during PND14-16 (*top*) along with representative immunoblot for FMRP distribution (*below*). **(B)** Line graph showing representative percentage FMRP distribution in polysomes during PND21-23 (*top*) and the corresponding representative immunoblot for FMRP distribution (*below*). **(C)** Bar graph showing FMRP distribution in translating/non-translating fractions in HET normalized to WT during PND14-16 (WT: *N* = 4; HET: *N* = 4) and PND21-23 (WT: *N* = 4; HET: *N* = 5). ^∗^*p* < 0.05 and ^∗∗^*p* < 0.01; Unpaired Student’s *t*-test. **(D)** Bar graph depicting relative *Fmr1* mRNA in translating fractions of HET normalized to WT during PND14-16 (WT: *N* = 3; HET: *N* = 3) and PND21-23 (WT: *N* = 3; HET: *N* = 5). ^∗^*p* < 0.05, NS, not significant; Unpaired Student’s *t*-test.

To understand whether the changes in the levels of translating *Syngap1* mRNA is a result of the altered association of FMRP with polysomes, we estimated the distribution of FMRP in translating/non-translating fraction from polysome profiling. We observed that the distribution of FMRP was increased in PND14-16 in the polysomal fraction in *Syngap1*^-/+^ (0.41 ± 0.03) as compared to age-matched WT (0.17 ± 0.02; *p* = 0.0011; Figure 4A,B,C). However, we observed reduced FMRP distribution in the polysomal fraction of *Syngap1*^-/+^ (0.23 ± 0.03) in PND21-23 compared to WT (0.55 ± 0.15; *p* = 0.0473; Figure 4A,B,C). That might have a compounding effect on the translation of FMRP target mRNAs during PND21-23 in *Syngap1*^-/+^ as the overall FMRP level was also reduced. As a control, we analyzed the PSD-95 levels during PND14-16 (WT = 2.03 ± 0.35; *Syngap1*^-/+^ = 1.39 ± 0.15; *p* = 0.125) and PND21-23 (WT = 0.98 ± 0.05; *Syngap1*^-/+^ = 0.98 ± 0.12; *p* = 0.96). However, we did not observe any change between WT and *Syngap1*^-/+^ (Supplementary Figure S4A).

Our previous result showed reduced FMRP level at PND21-23 in *Syngap1*^-/+^ as compared to its WT counterpart (Figure 1B). We investigated whether the reduced level of FMRP is due to altered *Fmr1* mRNA levels or translation. We evaluated the levels of *Fmr1* mRNA from the hippocampal lysates of WT and *Syngap1*^-/+^ mice at PND14-16 and PND21-23. We did not observe any significant difference in *Fmr1* mRNA levels in PND14-16 (WT = 0.019 ± 0.008; *Syngap1*^-/+^ = 0.029 ± 0.008; *p* = 0.4065) and PND21-23 (WT = 0.009 ± 0.001; *Syngap1*^-/+^ = 0.020 ± 0.009; *p* = 0.3129) between WT and *Syngap1*^-/+^ (Supplementary Figure S4B). This result suggests that reduction in FMRP levels in *Syngap1*^-/+^ mice at PND21-23 could be due to a decrease in *Fmr1* mRNA translation.

To further understand the translation status of *Fmr1* mRNA during PND14-16 and PND21-23, *Fmr1* mRNA present in translating fraction was quantified by qPCR. We found that *Fmr1* mRNA distribution in translating pool was unaltered in PND14-16 (WT = 66.66 ± 2.9%; *Syngap1*^-/+^ = 66.03 ± 4.1%; *p* = 0.9058) but was significantly reduced in *Syngap1*^-/+^ mice compared to WT in PND21-23 (WT = 89.34 ± 1.03%; *Syngap1*^-/+^ = 73.38 ± 4%; *p* = 0.0257; Figure 4D), indicating reduced FMRP level was a result of decreased *Fmr1* mRNA translation in PND21-23 in *Syngap1*^-/+^.

### Altered NMDAR-Mediated Translation Response in *Syngap1*^-/+^

Previous studies have shown increased levels of basal protein synthesis in *Syngap1*^-/+^ (Wang et al., 2013; Barnes et al., 2015). SYNGAP1 regulates synaptic maturation during a critical time window, and our results demonstrated altered expression of FMRP during a specific developmental stage in *Syngap1*^-/+^. Based on this, we hypothesized that the translational status could be different at these developmental stages. To study that, the phosphorylation status of eukaryotic Elongation Factor 2 (eEF2) was used as a read-out of translation response. Phosphorylation of eEF2 has been shown to repress global translation (Scheetz et al., 2000). We analyzed phospho/total-eEF2 in response to NMDAR stimulation from WT and *Syngap1*^-/+^ hippocampal synaptoneurosomes at PND14-16 and 21-23 using immunoblotting analysis. Hippocampal synaptoneurosome preparation was evaluated by validating the enrichment of PSD-95 as shown by Muddashetty et al., 2007 (Supplementary Figure S6A). As a proof of principle, we demonstrated that NMDAR stimulation of synaptoneurosomes from WT mice showed ∼1.5-fold increase in phospho/total-eEF2 1-min post-stimulation (Basal = 0.84 ± 0.11; Stimulated = 1.3 ± 0.12; *p* = 0.0376; Supplementary Figure S5A). To validate that the phosphorylation response of eEF2 is indeed resulting from NMDAR stimulation, we pre-treated the synaptoneurosomes with AP-5, a potent antagonist of NMDAR. The NMDAR-mediated phosphorylation was lost on AP-5 pre-treatment, showing the specificity of our assay (Supplementary Figure S6B).

**FIGURE 5:**
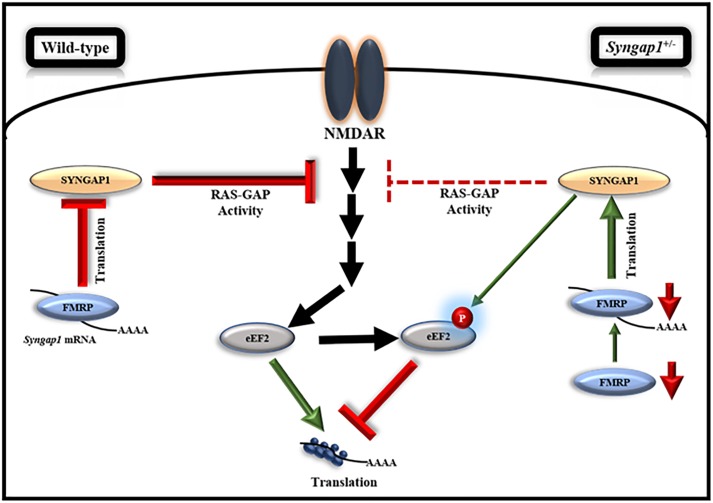
Model, illustrating the regulation of FMRP-mediated translation of *Syngap1* during development. This model shows that FMRP regulates *Syngap1* mRNA translation, which in turn regulates NMDAR-mediated signaling. In WT, NMDAR stimulation in synapse led to increased phosphorylation of eEF2, which resulted in global translation inhibition and the signaling was efficiently regulated by SYNGAP1. Whereas, in *Syngap1^-/+^* at PND14-16, NMDAR-mediated signaling was impaired as depicted by the loss of phosphorylation response to eEF2 due to a decreased level of SYNGAP1. At PND21-23 in *Syngap1^-/+^*, FMRP level was low that increased translation of *Syngap1* mRNA leading to an increased SYNGAP1 level compared to PND14-16. Thus, an elevated level of SYNGAP1 might recover the NMDAR-mediated signaling via phosphorylation of eEF2.

Further, we evaluated the translation response on NMDFR activation during development in *Syngap1*^-/+^. Our data showed an increase in phospho/total-eEF2 in *Syngap1*^-/+^ as compared to WT under basal conditions in both PND14-16 (WT = 0.84 ± 0.11; *Syngap1*^-/+^ = 1.6 ± 0.22%; *p* = 0.0245) and PND21-23 (WT = 0.22 ± 0.01%; *Syngap1*^-/+^ = 0.9 ± 0.11%; *p* = 0.0233 with Welch’s correction; Supplementary Figures S5B,C). We found that, at PND14-16, NMDAR-mediated increase in phosphorylation of eEF2 was not observed in synaptoneurosomes from *Syngap1*^-/+^ (stimulated/basal values for WT = 1.57 ± 0.2; *Syngap1*^-/+^ = 0.7 ± 0.09; *p* = 0.0069). Further analysis of this data by normalizing to WT showed a significant reduction in phospho/total eEF2 on NMDAR stimulation in *Syngap1*^-/+^ synaptoneurosomes (stimulated/basal values for WT = 1.00 ± 0.12; *Syngap1*^-/+^ = 0.45 ± 0.06; Supplementary Figure S5B). We hypothesize that it could be due to the increased level of phosphorylated-eEF2 at basal level in *Syngap1*^-/+^ mice. Surprisingly, even though we observed an increase in the level of phospho/total-eEF2 under the basal condition in PND21-23 in *Syngap1*^-/+^ synaptoneurosomes, NMDAR-mediated increase in phosphorylated-eEF2 in *Syngap1*^-/+^ was recovered to WT level (stimulated/basal values for WT = 1.81 ± 0.14; *Syngap1*^-/+^ = 1.92 ± 0.45; *p* = 0.8233; Supplementary Figure S5C). A similar phenomenon was observed in the 2-min stimulation of NMDAR (Supplementary Figures S6C,D). To verify that the loss in the NMDAR-mediated responses on eEF2 phosphorylation at PND14-16 in *Syngap1*^-/+^ is not due to unhealthy synaptoneurosomes, we measured the phosphorylated form of ERK, another well-known marker for NMDAR-mediated signaling pathway. We observed an increase in the phosphorylation of ERK upon NMDAR stimulation in both WT and *Syngap1*^-/+^ at PND14-16 (Supplementary Figure S5D), showing that the synaptoneurosomes were healthy. To further asses if the rescue in NMDAR-mediated signaling persists till adulthood, we performed similar experiments with PND > 60 mice. We further showed that NMDAR-mediated response on eEF2 phosphorylation was indeed absent in the *Syngap1*^-/+^ at PND > 60 (Supplementary Figure S6E). These data suggest that the rescue in NMDAR-mediated phosphorylation of eEF2 was transient and only present at a specific age window when FMRP is downregulated in *Syngap1*^-/+^ (Figure 5).

## Discussion

Many synaptic plasticity mechanisms are dependent on activity mediated local protein synthesis in neurons (Klann et al., 2004; Pfeiffer and Huber, 2006). Protein synthesis is regulated stringently in the synapse. One such crucial regulator of synaptic protein synthesis is FMRP, which is encoded by *FMR1* gene, the absence of which leads to Fragile X Syndrome, a monogenic cause of ID similar to *SYNGAP1*^-/+^ (Garber et al., 2008; Hamdan et al., 2009). Our observation of enhanced mGluR-LTD in the CA1 hippocampal region of *Syngap1*^-/+^ complements previous observation of enhanced basal protein synthesis in *Syngap1*^-/+^ prompted us to investigate the role of FMRP in the pathophysiology of *Syngap1*^-/+^ mutation (Wang et al., 2013; Barnes et al., 2015). Till date, only one report has studied interrelation between SYNGAP1 and FMRP (Barnes et al., 2015). They proposed that mutations in *Fmr1* and *Syngap1* lead to an opposite effect on synapse development, with FMRP deficits resulting in delayed synaptic maturation and deficit in SYNGAP1 causing accelerated maturation of dendritic spines. Considering this, Barnes et al. crossed *Fmr1*^-/Y^ with *Syngap1*^-/+^ but failed to rescue the neurophysiological deficits observed in *Syngap1*^-/+^ (Barnes et al., 2015). This study indicates that chronic depletion of these genes may not be a useful measure to rescue the pathophysiology observed in *Syngap1*^-/+^, as both these genes are essential for normal brain development. Since SYNGAP1 is known to regulate synaptic maturation during a specific developmental window (Clement et al., 2012, 2013), we hypothesized that the role of FMRP in *Syngap1*^-/+^ could also be developmentally regulated. Hence, we looked at the developmental expression profile of FMRP in the hippocampus of *Syngap1*^-/+^ mice. Our results show reduced expression of FMRP specifically in PND21-23 in *Syngap1*^-/+^. A study by Darnell et al., have identified *Syngap1* mRNA as one of the targets of FMRP by a high-throughput analysis. However, many such targets were not validated (Darnell et al., 2011). Our study is the first to validate the interaction between FMRP and *Syngap1* mRNA, thereby, regulating its translation. Our result suggests that the reduction in FMRP levels, as well as its reduced interaction with *Syngap1* mRNA at PND21-23 in *Syngap1*^-/+^, might lead to the compensatory increase in SYNGAP1 levels via increased *Syngap1* mRNA translation. In polysome profiling assay, we did not observe any significant difference in the A_254_ traces or the distribution of protein RPLP0 between WT and *Syngap1*^-/+^ animals indicating no difference in the basal translation in the hippocampus from *Syngap1*^-/+^ animals at PND14-16 and PND21-23.

Studies have reported that NMDAR-mediated signaling is dysregulated in *Syngap1*^-/+^ (Komiyama et al., 2002; Rumbaugh et al., 2006; Carlisle et al., 2008). These studies have further shown that SYNGAP1 associates with NR2B (Rockliffe and Gawler, 2006) and negatively regulates NMDAR-mediated ERK activation (Kim et al., 2005) and, hence, regulates insertion of AMPAR in the post-synaptic membrane (Rumbaugh et al., 2006). In line with this, Komiyama et al., have demonstrated increased basal levels of ERK phosphorylation in *Syngap1*^-/+^ (Komiyama et al., 2002) which does not explain the deficits observed in NMDAR-LTP in *Syngap1*^-/+^ mice as NMDAR stimulation resulted in a robust increase in ERK activation in slices from *Syngap1*^-/+^ mice (Komiyama et al., 2002). Thus, to understand the deficits seen in NMDAR-mediated signaling in *Syngap1*^-/+^ mice, we studied NMDAR-mediated translation repression. It has already been reported that NMDAR activation causes a reduction in global translation through phosphorylation of eEF2 (Scheetz et al., 2000). In our study, we measured the basal levels of phosphorylated eEF2 in hippocampal synaptoneurosomes from WT and *Syngap1*^-/+^ at PND14-16 and PND21-23 which showed increased phosphorylation of eEF2 at the basal condition in *Syngap1*^-/+^. This increase in the basal level of phosphorylation of eEF2 could be due to enhanced excitatory neuronal activity in *Syngap1*^-/+^ which might lead to an increase in Ca^2+^ levels and a subsequent increase in eEF2 phosphorylation via Ca^2+^-Calmodulin kinase. We report that, at PND14-16, NMDAR activation fails to cause eEF2 phosphorylation in *Syngap1*^-/+^ animals. Strikingly, even though we observed an increase in basal phospho/total-eEF2 in *Syngap1*^-/+^ synaptoneurosomes at PND21-23, NMDAR-mediated increase in eEF2 phosphorylation was similar to WT. This observation suggests that NMDAR-mediated translation response at PND21-23 in *Syngap1*^-/+^ may be restored. This change observed in PND21-23 could be due to a compensatory mechanism through increased NMDAR-mediated signaling. These findings further corroborate with the observations made by Clement et al. in which they have demonstrated increased synaptic transmission and increased AMPAR/NMDAR-mediated currents in PND14-16 but return to normal level in the later age (Clement et al., 2012). Based on our findings, we propose a model in which increased NMDAR-mediated response to protein synthesis is compensating for the loss of SYNGAP1 during development in *Syngap1*^-/+^. We further propose that fine-tuned downregulation of *Fmr1* translation during a specific developmental window in *Syngap1*^-/+^ mice might compensate for the dysregulation in NMDAR-mediated signaling.

These findings are interesting concerning the critical period of maturation of the hippocampus in mice. Early maturation of hippocampal neurons has been shown in *Syngap1*^-/+^ at PND14-16, whereas WT matures at PND21 (Clement et al., 2012). Our findings indicate that these two age groups are crucial for any compensation to occur. Once the window of critical period of development is lost, rescuing the pathophysiology becomes difficult.

Our data based on eEF2 phosphorylation on NMDAR activation is correlative to FMRP downregulation in *Syngap1*^-/+^ at PND21-23. Previous studies have shown dysregulated NMDAR-mediated signaling in the *Fmr1* KO mouse model owing to the fact that FMRP plays an essential role in NMDAR-mediated pathway (Toft et al., 2016). Also, whisker stimulation and visual experience that dependent on NMDAR activation led to increased FMRP protein level (Todd et al., 2003; Gabel et al., 2004). Therefore, NMDAR-mediated protein synthesis could be regulated by the level of FMRP as studies have shown that FMRP regulates translation downstream of NMDAR-mediated signaling (Chmielewska et al., 2018). However, regulation of NMDAR-mediated signaling proteins by FMRP in *Syngap1*^-/+^ is unclear. Our study is the first to suggest a potential regulation of NMDAR-mediated signaling proteins by FMRP. Thus, it is crucial to study FMRP’s role in NMDAR-mediated signaling and its regulation by FMRP.

## Conclusion

In conclusion, our study suggests that an altered response to activity-mediated protein synthesis during development is one of the major causes of abnormal neuronal function in *Syngap1*^-/+^. However, chronic depletion of two genes with common core pathophysiology may not be a useful measure to rescue the deficits observed in either of these mutations, i.e., *Fmr1^-/y^* and *Syngap1*^-/+^, as both these genes are essential for healthy brain development. Therefore, modulating these proteins at a specific developmental window could be a potential therapeutic strategy for treating ID-related pathophysiology.

## Ethics Statement

This study was carried out in accordance with Institutional Animal Ethics Committee (IAEC), and Committee for Purpose of Control and Supervision of Experiments on Animals (CPCSEA).

## Author Contributions

AP and BN performed all the experiments. JPC did mGluR-LTD in Figure 1A. SS performed part of the experiments in Figure 1B,C. DS did experiments in Figure 1B,C and part of the experiments in Supplementary Figures S1A,B, and S5D. AP, BN, RM, and JPC designed the experiments and wrote the manuscript. BN, SS, DS, RM and JPC edited the manuscript.

## Conflict of Interest Statement

The authors declare that the research was conducted in the absence of any commercial or financial relationships that could be construed as a potential conflict of interest.

## Footnotes

1 http://www.jax.org/strain/008890s

## Supplementary Material

FIGURE S1 FMRP and SYNGAP1 expression during development. **(A)** Representative Immunoblots for FMRP in WT at PND7-9, PND14-16, PND21-23, and PND > 60, normalized to β-ACTIN, and GAPDH (*top*). Line graph shows the expression profile of FMRP normalized to β-ACTIN (*middle*), and normalized to GAPDH (*below*) at PND7-9, PND14-16, PND21-23, and PND > 60 (*N* = 4 for all age groups, samples were run on the same gel); FMRP/ β-ACTIN: One-way ANOVA followed by Tukey’s multiple comparison test; PND7-9 vs. PND > 60: ^∗∗^*p* = 0.0084; PND14-16 vs. PND > 60: ^∗∗∗^*p* = 0.0007; PND21-23 vs. PND > 60: ^∗^*p* = 0.036. FMRP/GAPDH: One-way ANOVA followed by Tukey’s multiple comparison tests; PND7-9 vs PND > 60: ^∗∗^*p* = 0.0022; PND14-16 vs. PND > 60: ^∗∗^*p* = 0.0075. **(B)** Representative Immunoblots for SYNGAP1 in WT and HET at PND7-9, PND14-16, PND21-23, and PND > 60, normalized to β-ACTIN, and GAPDH (*top*). The line graph shows the expression profile of SYNGAP1 normalized to β-ACTIN (*middle*) and normalized to GAPDH (*below*; only WT) at PND7-9, PND14-16, PND21-23, and PND > 60 (*N* = 4 for all age groups, samples were run on the same gel). SYNGAP1/ β-ACTIN: Two-way ANOVA followed by Bonferroni’s multiple comparison test; WT vs HET at PND7-9: *p* = 0.23; PND14-16: ^∗∗^*p* = 0.0014; PND21-23: ^∗∗∗^*p* = 0.0006; PND > 60: ^∗∗∗^*p* = 0.0004. SYNGAP1/GAPDH: One-way ANOVA followed by Tukey’s multiple comparison tests; NS, not significant across age. **(C)** Multiple putative G-quadruplex was detected using QGRS Mapper in the validated sequence available for mouse *Syngap1* from NCBI (Gene ID: 240057). Three G- quadruplex sequences having high G-score were highlighted in the red box. All these sequences have been mapped in the Coding Sequence (CDS) (*left panel*). Multiple sequence alignment of the highest score G-quadruplexes of mouse *Syngap1* compared with Human and Rat. G score: 82 showing putative G-quadruplexes conserved among Human, Mouse, and Rat, respectively (*right panel*).

FIGURE S2 FMRP interacts with *Syngap1* mRNA in the hippocampus. **(A)** Bar graph showing relative mRNA enrichment in FMRP IP pellet compared to supernatant from the hippocampus of WT at PND14-16 normalized to IgG IP. Enrichment was calculated by the given formula: 2^-(dCtFMRPIP)^/2^-(dCtIgGIP)^; dCt = Ct (pellet) – Ct (Supernatant); *N* = 1. **(B)** Bar graph showing relative *Psd-95* mRNA enrichment in FMRP IP pellet compared to supernatant from hippocampus at PND14-16 (WT: *N* = 7; HET: *N* = 3) and PND21-23 (WT: *N* = 5; HET: *N* = 4) normalized to WT. Unpaired Student’s *t*-test. NS, not significant. **(C)** Representative immunoblot for SYNGAP1 and FMRP showing the expression of SYNGAP1 in transfected (T) compared to Un-transfected (UT) control. **(D)** Representative images of Hela cells showing the expression of GFP-SYNGAP1 (Green). Cell nuclei are stained with DAPI (Blue). The right panel shows a higher magnification image where GFP-SYNGAP1 shows punctate structure. **(E)** Representative immunoblot for FMRP normalized to β-ACTIN (*top*). The bar graph (*below*) shows a reduced level of FMRP in the *FMR1* siRNA treated cells compared to *scr* siRNA treated control (WT: *N* = 4; HET: *N* = 4). Unpaired Student’s *t*-test; ^∗^*p* < 0.05.

FIGURE S3 RPLP0 distribution unaltered in polysomes. **(A)** *β-actin* mRNA distribution in polysomes treated with cycloheximide and puromycin. **(B)** Representative percentage distribution of 18S rRNA in the polysome fractions of Cycloheximide and Puromycin treated WT samples in PND14-16. **(C)** Percentage distribution of 18S rRNA in the translating (Fractions 7–11) and non-translating (Fractions 1-6) pool of Cycloheximide and Puromycin treated WT samples in PND14-16. **(D)** Bar diagram showing *β-actin* mRNA distribution in Cycloheximide treated polysome HET normalized to WT in PND14-16 (WT: *N* = 6; HET: *N* = 6) and PND21-23 (WT: *N* = 4; HET: N = 5). NS, not significant. Unpaired Student’s *t*-test. **(E)** Representative percentage RPLP0 distribution line graph in PND14-16 (*left*) and PND21-23 (*right*). **(F)** Polyribosome profile obtained from Puromycin treated hippocampal lysate at PND14-16 in WT.

FIGURE S4 PSD-95 level in PND14-16 and PND21-23. **(A)** Representative immunoblots for PSD-95 normalized to β-ACTIN in the hippocampus during PND14-16 and PND21-23 in WT and HET. *Below* Bar graph showing a no significant difference in the level of PSD-95 at PND14-16 (WT: *N* = 6; HET: *N* = 6) and PND21-23 (WT: *N* = 7; HET: *N* = 4) between WT and HET; NS = not significant. Unpaired Student’s *t*-test. **(B)** Bar graph depicting relative *Fmr1* mRNA normalized to *β-actin* from total hippocampal lysate at PND14-16 (*left*, WT: *N* = 3; HET: *N* = 3) and PND21-23 (*right*, WT: *N* = 3; HET: *N* = 3); NS, not significant. Unpaired Student’s *t*-test.

FIGURE S5 Dysregulated NMDAR-mediated translation response is recovered during PND21-23 in HET. **(A)** Representative immunoblot for Phospho-eEF2 and Total-eEF2 showing increased phosphorylation on NMDAR stimulation for 1-min in synaptoneurosomes from WT during PND21-23 (*left*). Pooled data of the same represented in the bar graph (*right*, Basal: *N* = 4; Stimulated: *N* = 4); ^∗^*p* < 0.05; Unpaired Student’s *t*-test. **(B)** Representative immunoblots of phospho- and total-eEF2 normalized to β-ACTIN during PND14-16 in WT and HET (*top*). The bar graph shows increased phosphorylation of eEF2 at basal conditions in synaptoneurosome obtained from the hippocampus of HET as compared to WT during PND14-16 in the (*middle*, WT: *N* = 4; HET: *N* = 3). Bar graph showing decreased phosphorylation of eEF2 in HET on NMDAR stimulation as compared to WT in PND14-16 (*below*, WT: *N* = 4; HET: *N* = 4). ^∗^*p* < 0.05, ^∗∗^*p* < 0.01; Unpaired Student’s *t*-test; WT and HET samples were run on the same gel; 1-min stimulation blots were cropped and showed here. **(C)** Representative immunoblots for phospho-eEF2 and total-eEF2 normalised to β-ACTIN during PND21-23 in WT and HET (*top*). Increased phosphorylation of eEF2 at the basal condition in HET synaptoneurosome as compared to WT during PND21-23 (*middle*, WT: *N* = 3; HET: *N* = 3). Bar graph showing the extent of phosphorylation in HET is similar to WT during PND21-23 (*bottom*, WT: *N* = 3; HET: *N* = 4). ^∗^*p* < 0.05, NS, not significant; Unpaired Student’s *t*-test. **(D)** Representative immunoblot for Phospho-ERK1/2 and Total-ERK1/2 showing increased phosphorylation on NMDAR stimulation for 5-min in synaptoneurosomes from WT and HET during PND14-16 (*left*). Pooled data of the same represented in the bar graphs (*right*, Basal: *N* = 4; Stimulated: *N* = 4); ^∗^*p* < 0.05; Unpaired Student’s *t*-test.

FIGURE S6 Altered phosphorylation of eEF2 in HET. **(A)** Representative immunoblot depicting the enrichment of PSD-95 in synaptoneurosome (S) compared to Total hippocampal lysate (T; *top*). A bar graph is showing quantified data normalised to α-TUBULIN (*below*). **(B)** Representative immunoblot for Phospho-eEF2, Total-eEF2, and β-ACTIN in synaptoneurosomes after 1-min stimulation with NMDA, AP-5, and NMDA+AP-5 (*top*). Quantified data as histogram shows increased phosphorylation of eEF2 on NMDA treatment is lost when co-treated with AP-5 (*below*). ^∗^*p* < 0.05, One-way ANOVA followed by Dunnett’s multiple comparison tests. **(C)** Representative immunoblot images for Phospho-eEF2, Total-eEF2, and β-ACTIN in synaptoneurosomes after 1-min and 2-min NMDAR stimulation during PND14-16 (*top*). All samples (both WT and HET) were run on the same gel (B: Basal; N: NMDA stimulation). 1-min stimulation bands were cropped and represented in Supplementary Figure S5. Bar graph showing that a 2-min activation of NMDAR alters phosphorylation of eEF2 in HET (*N* = 3) compared to WT (*N* = 3) in PND14-16 (*below*). ^∗^*p* < 0.05; NS, not significant. Unpaired Student’s *t*-test. **(D)** Representative immunoblot for Phospho- and Total-eEF2 normalized to β-ACTIN synaptoneurosomes stimulated with NMDA for 2-min in WT and HET during PND21-23 (*top*). Bar graph depicting unaltered phosphorylation of eEF2 in HET (*N* = 3) compared to WT (*N* = 3) post-2-min activation of NMDAR. NS, not significant. Unpaired Student’s *t*-test. **(E)** Representative immunoblots of phospho- and total-eEF2 normalized to β-ACTIN during PND > 60 in WT and HET (*left*). Bar graph showing decreased phosphorylation of eEF2 in HET on NMDAR stimulation as compared to WT in PND > 60 (*right*, WT: *N* = 4; HET: *N* = 4). ^∗^*p* < 0.05; Unpaired Student’s *t*-test.
