# Supplementary figures and images for: Differential Regulation of Syngap1 Translation by FMRP Modulates eEF2 Mediated Response on NMDAR Activity
Source: Front Mol Neurosci. 2019 May 9;12:97. doi: 10.3389/fnmol.2019.00097 (PMC6520660; doi:10.3389/fnmol.2019.00097)

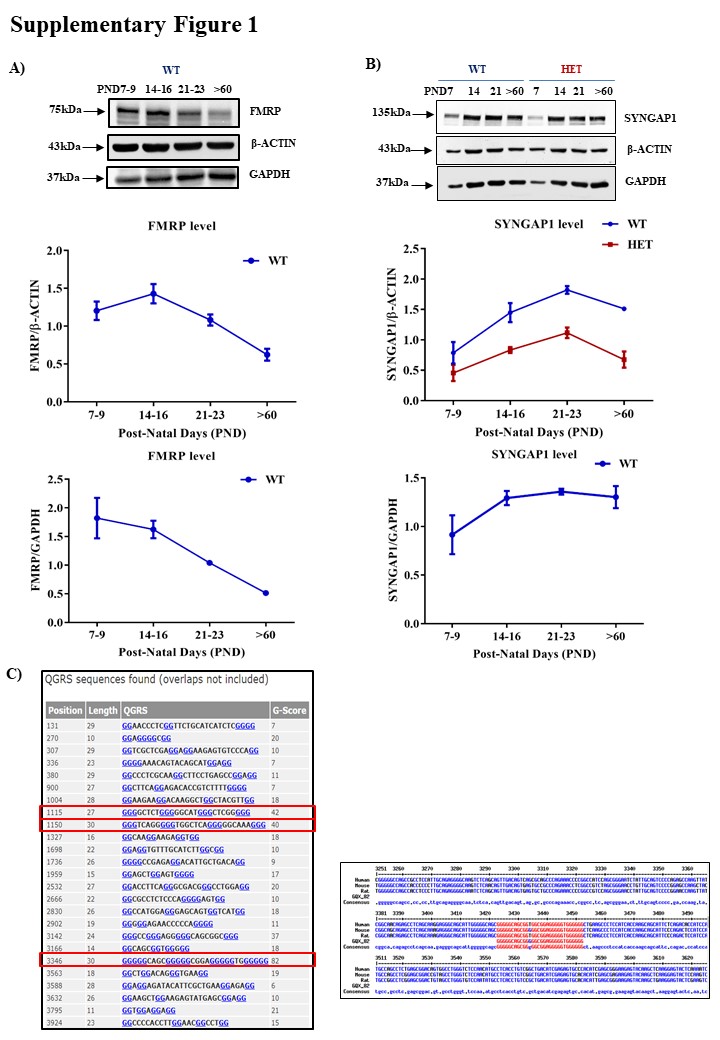

Supplement: FIGURE S1 — FMRP and SYNGAP1 expression during development. (A) Representative Immunoblots for FMRP in WT at PND7-9, PND14-16, PND21-23, and PND > 60, normalized to β-ACTIN, and GAPDH (top). Line graph shows the expression profile of FMRP normalized to β-ACTIN (middle), and normalized to GAPDH (below) at PND7-9, PND14-16, PND21-23, and PND > 60 (N = 4 for all age groups, samples were run on the same gel); FMRP/ β-ACTIN: One-way ANOVA followed by Tukey’s multiple comparison test; PND7-9 vs. PND > 60: ∗∗p = 0.0084; PND14-16 vs. PND > 60: ∗∗∗p = 0.0007; PND21-23 vs. PND > 60: ∗p = 0.036. FMRP/GAPDH: One-way ANOVA followed by Tukey’s multiple comparison tests; PND7-9 vs PND > 60: ∗∗p = 0.0022; PND14-16 vs. PND > 60: ∗∗p = 0.0075. (B) Representative Immunoblots for SYNGAP1 in WT and HET at PND7-9, PND14-16, PND21-23, and PND > 60, normalized to β-ACTIN, and GAPDH (top). The line graph shows the expression profile of SYNGAP1 normalized to β-ACTIN (middle) and normalized to GAPDH (below; only WT) at PND7-9, PND14-16, PND21-23, and PND > 60 (N = 4 for all age groups, samples were run on the same gel). SYNGAP1/ β-ACTIN: Two-way ANOVA followed by Bonferroni’s multiple comparison test; WT vs HET at PND7-9: p = 0.23; PND14-16: ∗∗p = 0.0014; PND21-23: ∗∗∗p = 0.0006; PND > 60: ∗∗∗p = 0.0004. SYNGAP1/GAPDH: One-way ANOVA followed by Tukey’s multiple comparison tests; NS, not significant across age. (C) Multiple putative G-quadruplex was detected using QGRS Mapper in the validated sequence available for mouse Syngap1 from NCBI (Gene ID: 240057). Three G- quadruplex sequences having high G-score were highlighted in the red box. All these sequences have been mapped in the Coding Sequence (CDS) (left panel). Multiple sequence alignment of the highest score G-quadruplexes of mouse Syngap1 compared with Human and Rat. G score: 82 showing putative G-quadruplexes conserved among Human, Mouse, and Rat, respectively (right panel). [file Image_1.jpg]

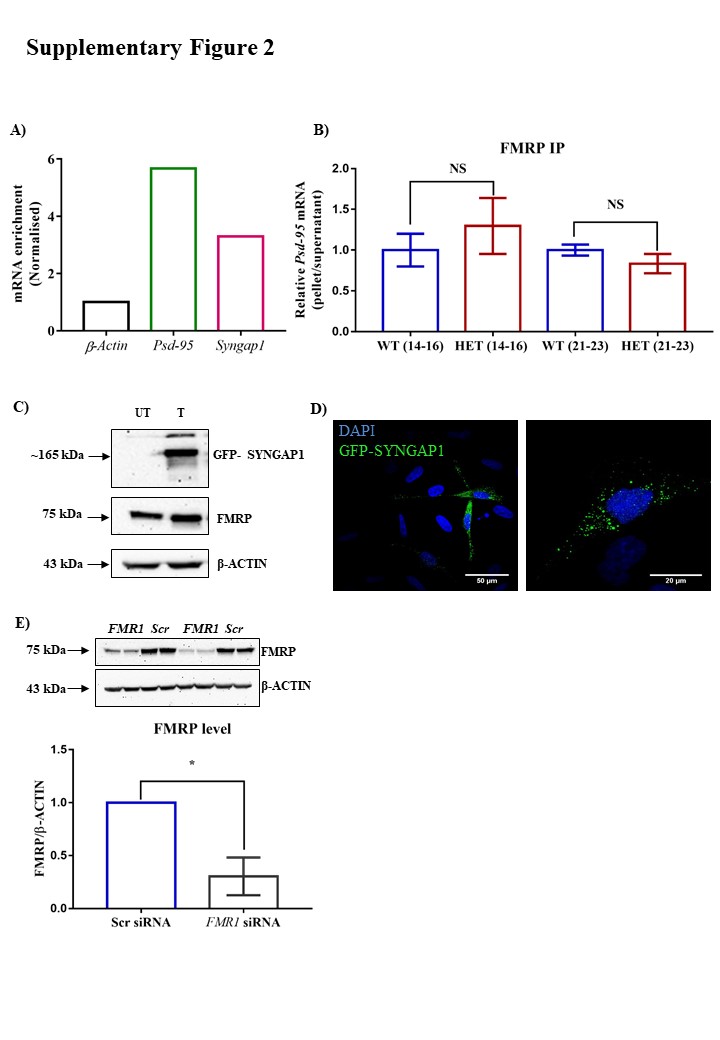

Supplement: FIGURE S2 — FMRP interacts with Syngap1 mRNA in the hippocampus. (A) Bar graph showing relative mRNA enrichment in FMRP IP pellet compared to supernatant from the hippocampus of WT at PND14-16 normalized to IgG IP. Enrichment was calculated by the given formula: 2-(dCtFMRPIP)/2-(dCtIgGIP); dCt = Ct (pellet) – Ct (Supernatant); N = 1. (B) Bar graph showing relative Psd-95 mRNA enrichment in FMRP IP pellet compared to supernatant from hippocampus at PND14-16 (WT: N = 7; HET: N = 3) and PND21-23 (WT: N = 5; HET: N = 4) normalized to WT. Unpaired Student’s t-test. NS, not significant. (C) Representative immunoblot for SYNGAP1 and FMRP showing the expression of SYNGAP1 in transfected (T) compared to Un-transfected (UT) control. (D) Representative images of Hela cells showing the expression of GFP-SYNGAP1 (Green). Cell nuclei are stained with DAPI (Blue). The right panel shows a higher magnification image where GFP-SYNGAP1 shows punctate structure. (E) Representative immunoblot for FMRP normalized to β-ACTIN (top). The bar graph (below) shows a reduced level of FMRP in the FMR1 siRNA treated cells compared to scr siRNA treated control (WT: N = 4; HET: N = 4). Unpaired Student’s t-test; ∗p < 0.05. [file Image_2.jpg]

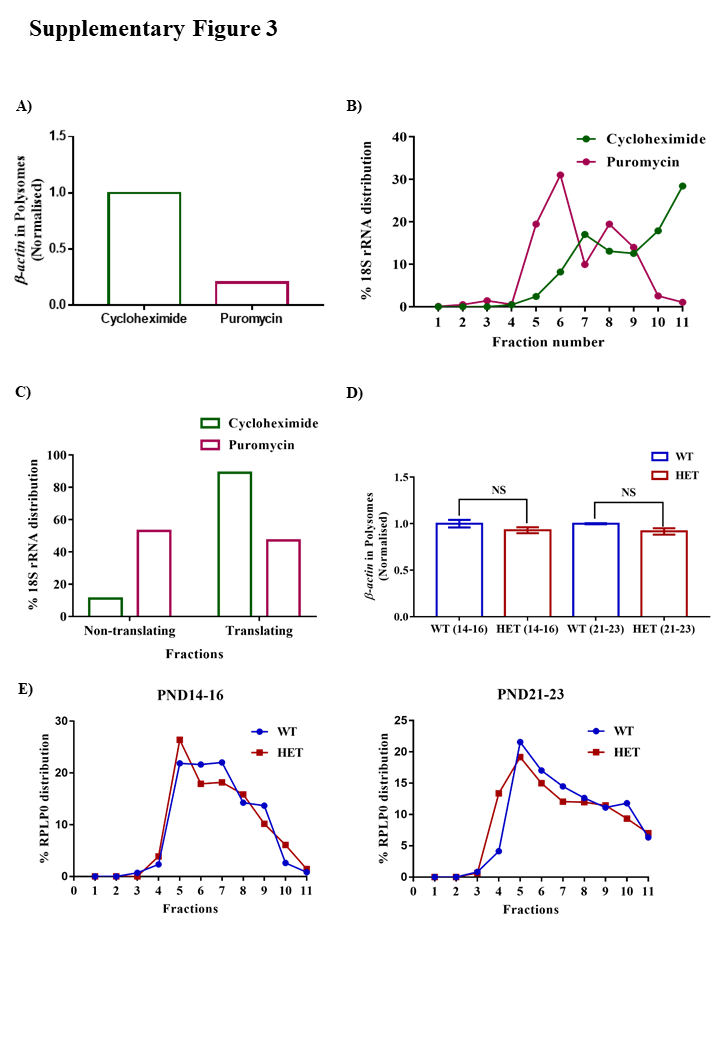

Supplement: FIGURE S3 — RPLP0 distribution unaltered in polysomes. (A) β-actin mRNA distribution in polysomes treated with cycloheximide and puromycin. (B) Representative percentage distribution of 18S rRNA in the polysome fractions of Cycloheximide and Puromycin treated WT samples in PND14-16. (C) Percentage distribution of 18S rRNA in the translating (Fractions 7–11) and non-translating (Fractions 1-6) pool of Cycloheximide and Puromycin treated WT samples in PND14-16. (D) Bar diagram showing β-actin mRNA distribution in Cycloheximide treated polysome HET normalized to WT in PND14-16 (WT: N = 6; HET: N = 6) and PND21-23 (WT: N = 4; HET: N = 5). NS, not significant. Unpaired Student’s t-test. (E) Representative percentage RPLP0 distribution line graph in PND14-16 (left) and PND21-23 (right). (F) Polyribosome profile obtained from Puromycin treated hippocampal lysate at PND14-16 in WT. [file Image_3.TIF]

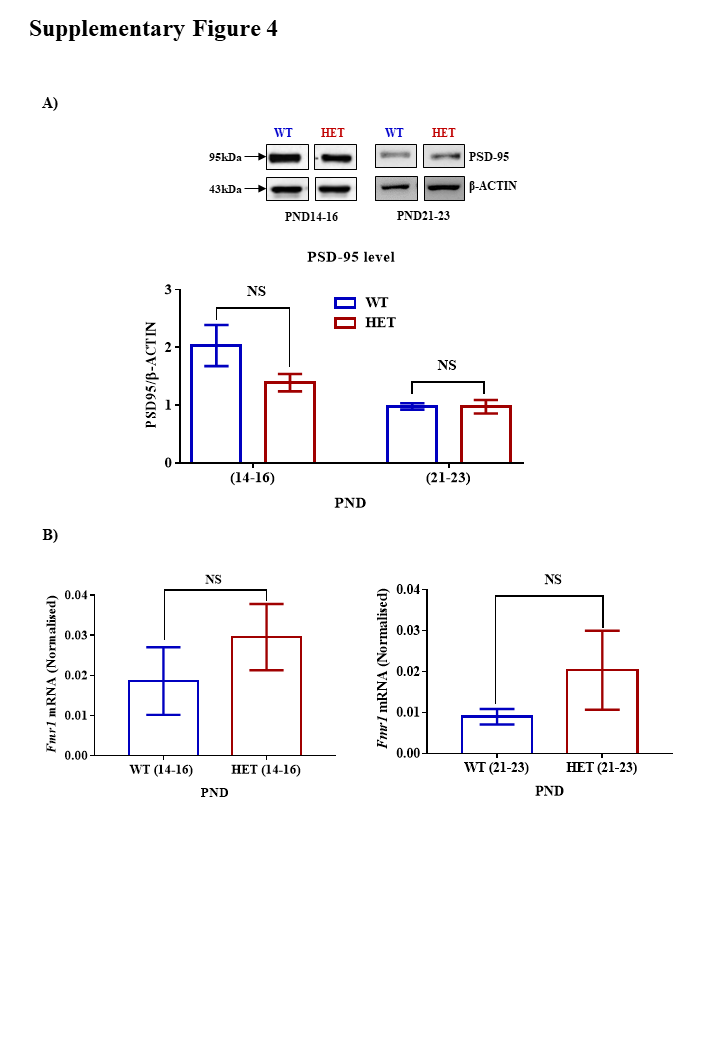

Supplement: FIGURE S4 — PSD-95 level in PND14-16 and PND21-23. (A) Representative immunoblots for PSD-95 normalized to β-ACTIN in the hippocampus during PND14-16 and PND21-23 in WT and HET. Below Bar graph showing a no significant difference in the level of PSD-95 at PND14-16 (WT: N = 6; HET: N = 6) and PND21-23 (WT: N = 7; HET: N = 4) between WT and HET; NS = not significant. Unpaired Student’s t-test. (B) Bar graph depicting relative Fmr1 mRNA normalized to β-actin from total hippocampal lysate at PND14-16 (left, WT: N = 3; HET: N = 3) and PND21-23 (right, WT: N = 3; HET: N = 3); NS, not significant. Unpaired Student’s t-test. [file Image_4.TIF]

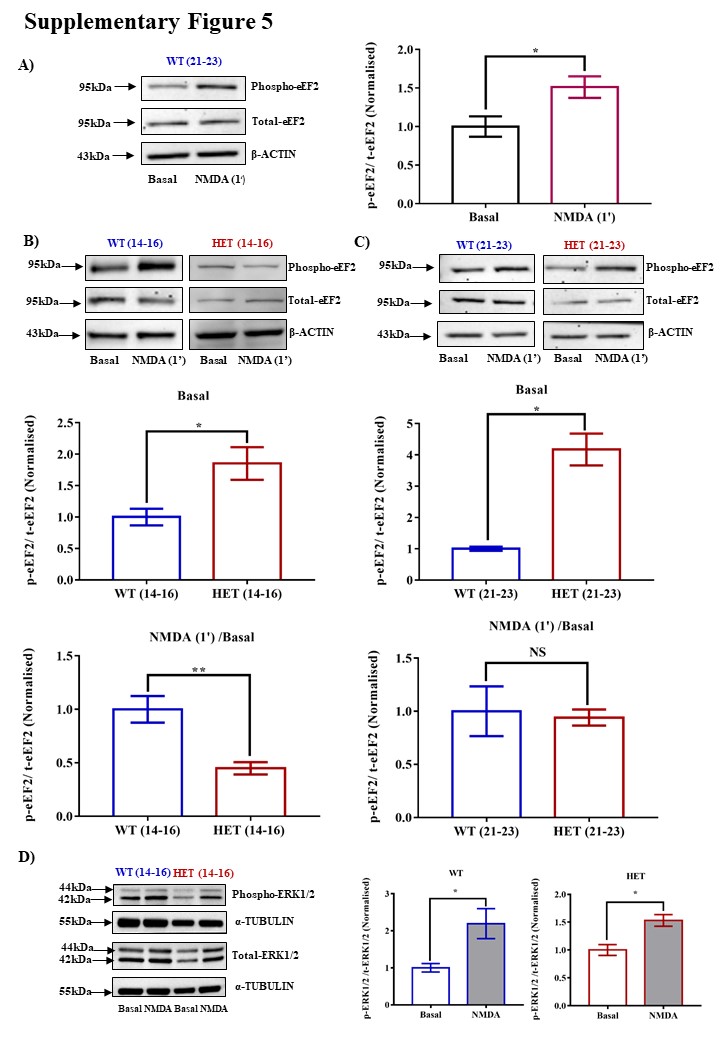

Supplement: FIGURE S5 — Dysregulated NMDAR-mediated translation response is recovered during PND21-23 in HET. (A) Representative immunoblot for Phospho-eEF2 and Total-eEF2 showing increased phosphorylation on NMDAR stimulation for 1-min in synaptoneurosomes from WT during PND21-23 (left). Pooled data of the same represented in the bar graph (right, Basal: N = 4; Stimulated: N = 4); ∗p < 0.05; Unpaired Student’s t-test. (B) Representative immunoblots of phospho- and total-eEF2 normalized to β-ACTIN during PND14-16 in WT and HET (top). The bar graph shows increased phosphorylation of eEF2 at basal conditions in synaptoneurosome obtained from the hippocampus of HET as compared to WT during PND14-16 in the (middle, WT: N = 4; HET: N = 3). Bar graph showing decreased phosphorylation of eEF2 in HET on NMDAR stimulation as compared to WT in PND14-16 (below, WT: N = 4; HET: N = 4). ∗p < 0.05, ∗∗p < 0.01; Unpaired Student’s t-test; WT and HET samples were run on the same gel; 1-min stimulation blots were cropped and showed here. (C) Representative immunoblots for phospho-eEF2 and total-eEF2 normalised to β-ACTIN during PND21-23 in WT and HET (top). Increased phosphorylation of eEF2 at the basal condition in HET synaptoneurosome as compared to WT during PND21-23 (middle, WT: N = 3; HET: N = 3). Bar graph showing the extent of phosphorylation in HET is similar to WT during PND21-23 (bottom, WT: N = 3; HET: N = 4). ∗p < 0.05, NS, not significant; Unpaired Student’s t-test. (D) Representative immunoblot for Phospho-ERK1/2 and Total-ERK1/2 showing increased phosphorylation on NMDAR stimulation for 5-min in synaptoneurosomes from WT and HET during PND14-16 (left). Pooled data of the same represented in the bar graphs (right, Basal: N = 4; Stimulated: N = 4); ∗p < 0.05; Unpaired Student’s t-test. [file Image_5.jpg]

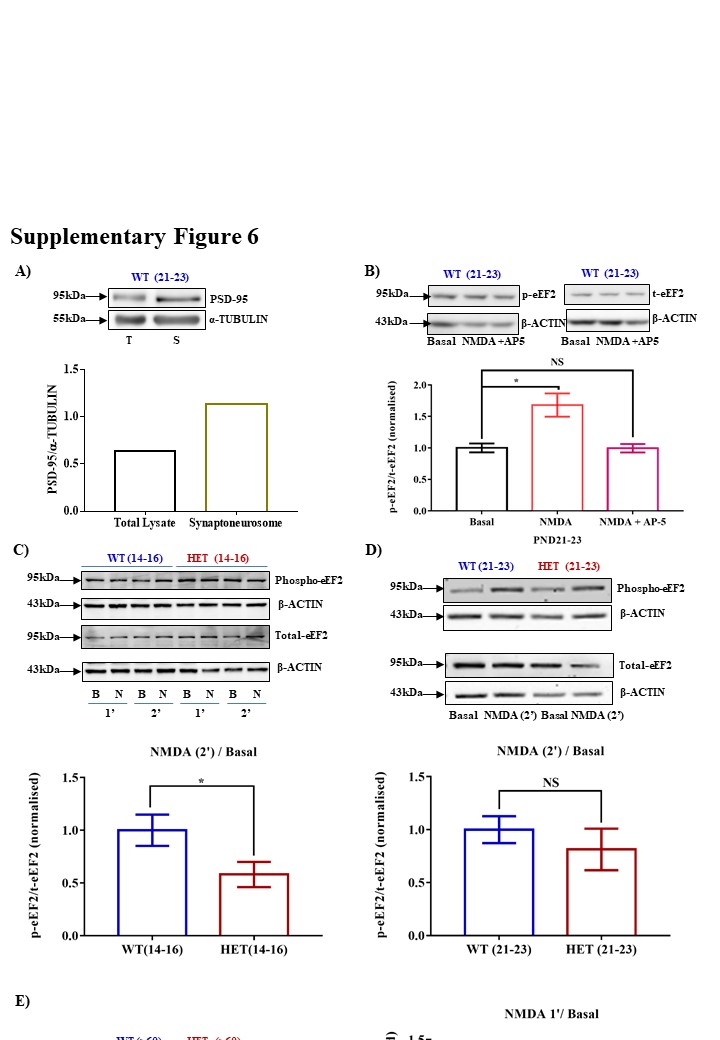

Supplement: FIGURE S6 — Altered phosphorylation of eEF2 in HET. (A) Representative immunoblot depicting the enrichment of PSD-95 in synaptoneurosome (S) compared to Total hippocampal lysate (T; top). A bar graph is showing quantified data normalised to α-TUBULIN (below). (B) Representative immunoblot for Phospho-eEF2, Total-eEF2, and β-ACTIN in synaptoneurosomes after 1-min stimulation with NMDA, AP-5, and NMDA+AP-5 (top). Quantified data as histogram shows increased phosphorylation of eEF2 on NMDA treatment is lost when co-treated with AP-5 (below). ∗p < 0.05, One-way ANOVA followed by Dunnett’s multiple comparison tests. (C) Representative immunoblot images for Phospho-eEF2, Total-eEF2, and β-ACTIN in synaptoneurosomes after 1-min and 2-min NMDAR stimulation during PND14-16 (top). All samples (both WT and HET) were run on the same gel (B: Basal; N: NMDA stimulation). 1-min stimulation bands were cropped and represented in Supplementary Figure S5. Bar graph showing that a 2-min activation of NMDAR alters phosphorylation of eEF2 in HET (N = 3) compared to WT (N = 3) in PND14-16 (below). ∗p < 0.05; NS, not significant. Unpaired Student’s t-test. (D) Representative immunoblot for Phospho- and Total-eEF2 normalized to β-ACTIN synaptoneurosomes stimulated with NMDA for 2-min in WT and HET during PND21-23 (top). Bar graph depicting unaltered phosphorylation of eEF2 in HET (N = 3) compared to WT (N = 3) post-2-min activation of NMDAR. NS, not significant. Unpaired Student’s t-test. (E) Representative immunoblots of phospho- and total-eEF2 normalized to β-ACTIN during PND > 60 in WT and HET (left). Bar graph showing decreased phosphorylation of eEF2 in HET on NMDAR stimulation as compared to WT in PND > 60 (right, WT: N = 4; HET: N = 4). ∗p < 0.05; Unpaired Student’s t-test. [file Image_6.jpg]
